# Big Data, Big Responsibility! Building best-practice privacy strategies into a large-scale neuroinformatics platform

**DOI:** 10.23889/ijpds.v1i1.364

**Published:** 2017-04-19

**Authors:** Christina Popovich, Francis Jeanson, Brendan Behan, Shannon Lefaivre, Aparna Shukla

**Affiliations:** 1 Ontario Brain Institute

## Objectives

The Ontario Brain Institute (OBI) has begun to catalyze scientific discovery in the field of neuroscience through its' large-scale informatics platform, known as Brain-CODE (Centre for Ontario Data Exploration). Brain-CODE manages the acquisition, storage, processing, and analytics of multidimensional data collected from patients with a variety of brain disorders. Our vision is for the platform to act as an informatics catalyst; encouraging multidisciplinary research collaboration, data integration, and innovation in neuroscience research. Brain-CODE's infrastructure was designed with best-practice privacy strategies built at the forefront to enable secure data capture of sensitive patient information in a manner that abides by government legislation while fostering data sharing and linking opportunities.

## Approach

Privacy and security features have been incorporated into the very foundation of Brain-CODE's comprehensive guidelines, which are reinforced by our state-of-the-art approaches to keep patient data safe. To ensure clarity for study participants, we have developed standard consent language outlining how sensitive patient data will be collected, entered, de-identified, and shared using Brain-CODE. Moreover, our tiered approach to data accessibility enables the storage of encrypted Ontario Health Card Numbers as well as other patient information, secure long-term storage of de-identified data, and data sharing opportunities by request from third parties following risk-based analysis re-identification techniques. OBI has also established a comprehensive Information Security Policy and Informatics Governance Policies, as well as a carried out a Privacy Impact Assessment and Threat Risk Assessment for Brain-CODE.

## Results

Brain-CODE is proudly named a "Privacy by Design" Ambassador by the Office of the Information and Privacy Commissioner of Ontario, Canada. Moreover, approximately 200 neuroscience researchers and 35 institutions from across Canada have adopted our standard consent language to enable secure data sharing within and across neurological disorders as well as linkage opportunities with national and international databases in a secure environment.

## Conclusion

OBI's rigorous approach to data sharing in the field of neuroscience maintains the accessibility of research data for big discoveries without compromising patient privacy and security. We believe that Brain-CODE is a powerful and advantageous tool; moving neuroscience research from independent silos to an integrative system approach for improving patient health. OBI's vision for improved brain health for patients living with neurological disorders paired with Brain-CODE's best-practice strategies in privacy protection of patient data offer a novel and innovative approach to "big data" initiatives aimed towards improving public health and society world-wide.

